# A deoxyviolacein‐based transposon insertion vector for pigmented tracer studies

**DOI:** 10.1002/mbo3.1425

**Published:** 2024-07-10

**Authors:** Benjamin R. Dietz, Tyler J. Nelson, Neil E. Olszewski, Brett M. Barney

**Affiliations:** ^1^ Department of Bioproducts and Biosystems Engineering University of Minnesota St. Paul Minnesota USA; ^2^ Department of Plant and Microbial Biology University of Minnesota St. Paul Minnesota USA; ^3^ Biotechnology Institute University of Minnesota St. Paul Minnesota USA

**Keywords:** deoxyviolacein, endophyte, pigment production, screening, transposon

## Abstract

Pigments provide a simple means to rapidly visually ascertain the quantities or presence of specific microbes in a complex community. The selection of pigment‐producing colonies that are simple to differentiate from common colony phenotypes provides a high degree of certainty for the identity of pigment‐tagged strains. Successful employment of pigment production is dependent on various intrinsic factors related to proper levels of gene expression and pigment production that are not always easy to predict and vary within each microbe. We have constructed a simple transposon system that incorporates the genes for the production of deoxyviolacein, a pigment produced from intracellular reserves of the amino acid tryptophan, to randomly insert these genes throughout the genome. This tool allows the user to select from many thousands of potential sites throughout a bacterial genome for an ideal location to generate the desired amount of pigment. We have applied this system to a small selection of endophytes and other model bacteria to differentiate these strains from complex communities and confirm their presence after several weeks in natural environments. We provide two examples of applications using the pigments to trace strains following introduction into plant tissues or to produce a reporter strain for extracellular nitrogen compound sensing. We recognize that this tool could have far broader utility in other applications and microbes, and describe the methodology for use by the greater scientific community.

## Graphical abstract

An example experiment using deoxyviolaceint‐based transposon insertion vectors to transfect a target microbe. Differences in pigment production are easily screened for further downstream experiments.

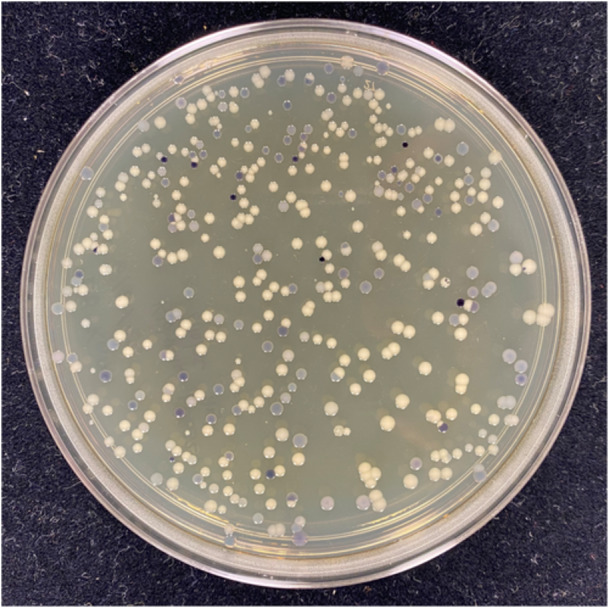

## 1 INTRODUCTION

Visual selective markers that generate either a pigment or a fluorescent protein are useful to identify or track specific microbes, especially when the microbe is a component of a complex community, where differences between various microbes are not inherently obvious. Transposon mutagenesis is a powerful tool that enables one to randomly integrate a specific DNA fragment into a bacterial genome to generate large mutant libraries (Hayes, 2003). These mutant libraries can then be used in a variety of downstream processes, though the libraries are commonly used to search for specific phenotypes that are associated with the disruption of specific genes (Barney et al., 2015; Hayes, 2003). An antibiotic resistance gene is generally used as a selection marker, as it allows for the isolation of clones that carry the desired transposon following conjugation.

The Mariner transposon system (Rubin et al., 1999) randomly inserts into thiamine‐adenine adjacent sites (TA sites) within the DNA of the target bacterial genome. Our laboratory constructed two deoxyviolacein piggyback transposon vectors based on the Mariner transposon system. These two vectors differ only in the selectable marker (tetracycline or kanamycin). The deoxyviolacein‐operon transposon construct (Kittleson et al., 2012) carries a ribosomal binding site upstream of *vioA*, the first deoxyviolacein gene in the operon, but does not contain a known internal promoter to induce expression of the deoxyviolacein operon. In this manner, insertions into TA sites that do not contain a sufficient upstream promoter should result in minimal production of the deoxyviolacein pigment. However, in cases where the transposon inserts into a TA site with a suitable indigenous upstream promoter sufficient to induce expression of the deoxyviolacein cassette, colonies can be visualized by the accumulation of the chromogenic product. These constructs allow one to quickly survey a selection of several thousand clones to identify specific colonies with the desired level of pigment biosynthesis, without the need for specific knowledge about any indigenous promoter systems within the host strain.

In our laboratory, we used various transposon‐derived clones that produce pigments to track bacterial endophytes that are growing in association with plants. Following harvesting and disruption of the plant organs, the associate microbes can be plated on a standard medium, and tagged cells are differentiated from potential contaminants or other natural endophytes based on the production of the pigment. We further engineered a microbe to serve as a nitrogen‐compound‐dependent biosensor to identify microbes that produce elevated levels of extracellular ammonium or other nitrogen compounds. We envision many other applications where pigmented bacteria would be useful for identification, and recognize that this transposon might have many other uses beyond those employed in our research laboratories. We have provided these plasmid vectors through Addgene, and demonstrate their potential application in a variety of bacterial hosts, presuming that these hosts do not naturally produce deoxyviolacein or another pigment with a similar color.

## 2 MATERIALS AND METHODS

### 2.1 Genetic constructs of deoxyviolacein piggyback transposon vectors

**Figure 1:**
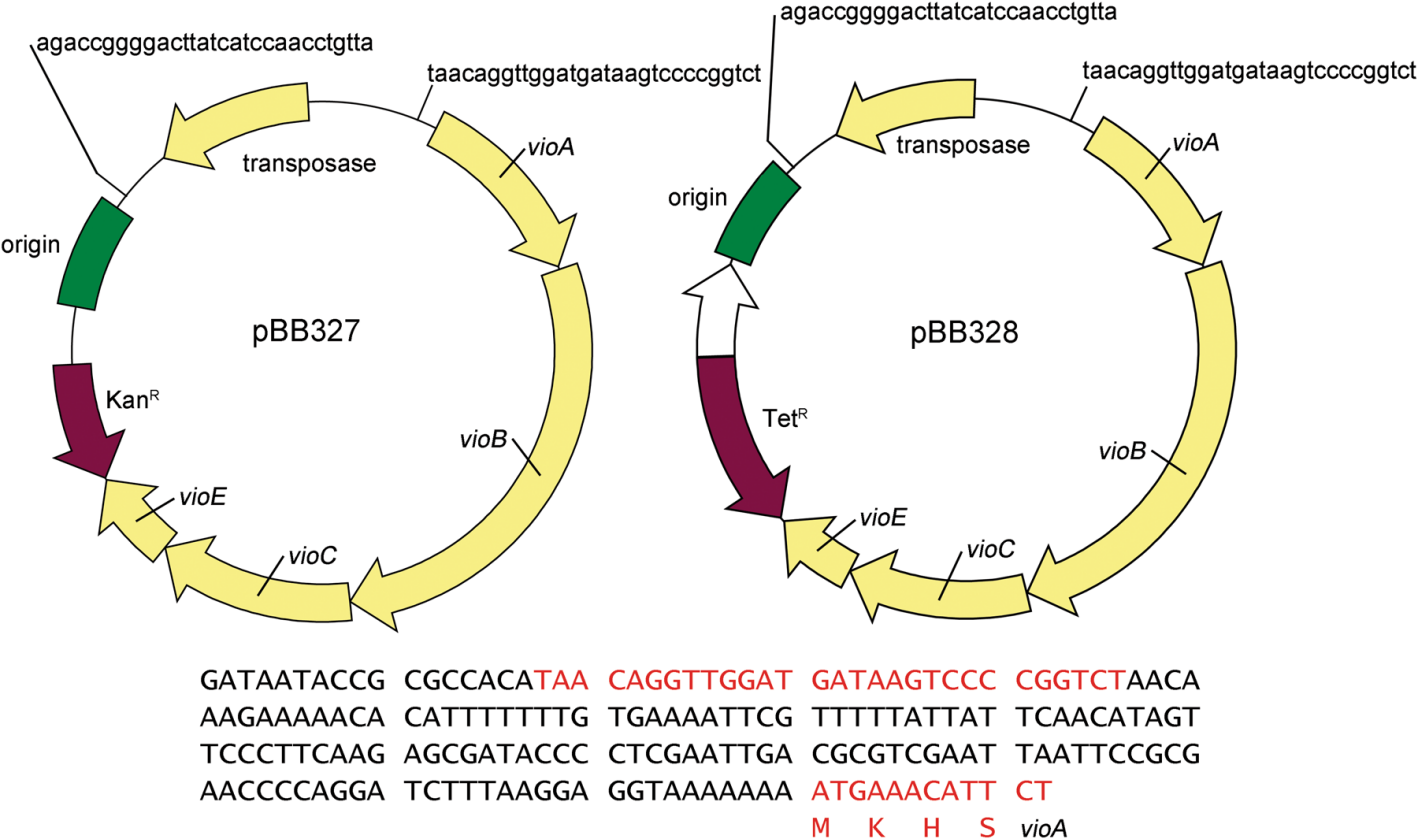
Plasmid illustrations. Shown above are plasmid maps showing annotations for various components of the deoxyviolacein‐based transposon constructs. Plasmid pBB327 is a kanamycin‐selectable vector. Plasmid pBB328 is a tetracycline‐selectable vector. Shown below is the sequence starting from the TA insertion to the ATG start codon of *vioA*.

Two plasmids were constructed to introduce deoxyviolacein biosynthetic genes through transposon mutagenesis. The deoxyviolacein operon was obtained from Christopher Anderson (Kittleson et al., 2012) through Addgene (BBa_J72214‐BBa_J72090), and was selected based on sufficient pigment production of the initial construct. When utilized, site‐directed mutagenesis was performed using the Quikchange II protocol (Agilent, Santa Clara, CA). Plasmids pBB327 and pBB328 were constructed as detailed in Table 1 and primers used to modify these are provided in Table 2. These plasmids are similar to one another, except for different antibiotic selection genes inside the transposon (kanamycin for pBB327 and tetracycline for pBB328). The plasmids contain a transposase, R6K origin, deoxyviolacein expression genes *vioABCE*, an antibiotic marker, and two repeat sites where the transposase will cut. A map of the plasmids is shown in Figure 1. All plasmid constructs were first introduced into and isolated from *Escherichia coli* BW25141 and then transformed into *E. coli* WM3064 for conjugation into recipient strains.

**Table 1:** Key plasmid constructs utilized in this work.

Plasmid^a^	Relevant gene(s) cloned or plasmids manipulated	Parent vector	Source or reference
pBB284	Moved deoxyviolacein cassette from BBa_J72214‐BBa_J72090 plasmid into pBB053 plasmid to make pUC19 derivative plasmid with deoxyviolacein selection for routine cloning.	BBa_J72214‐BBa_J72090 and pBB053	Barney et al. (2015), Kittleson et al. (2012)
pBB295	Performed site‐directed mutagenesis on pEB001 to add BamHI site.	pEB001	Brutinel and Gralnick (2012)
pBB296	Performed site‐directed mutagenesis on pBB295 to add BamHI site.	pBB295	This study
pBB298	Moved tetracycline cassette from pBBTET6 into pBB296.	pBB296 and pBBTET6	This study
pBB309	Performed site‐directed mutagenesis on pBB295 to replace PstI site with KpnI site.	pBB295	This study
pBB327	Moved deoxyviolacein cassette from pBB284 into pBB309.	pBB284 and pBB309	This Study
pBB328	Moved deoxyviolacein cassette from pBB284 into pBB298.	pBB284 and pBB298	This Study
pPCRNH3‐21	Construct to replace *nifLA* from *Azotobacter vinelandii* with tetracycline cassette	pPCRNH3‐13	Barney et al. (2015)

^a^ The sequences of all plasmids in this study are available upon request.

**Table 2:** Key primers used in this study.

Primer	Sequence 5′–3′	Purpose
BBP1176	GCTTTACTGG CACTTCAGGA ACAAGC	Sequencing Primer
BBP1282	GCGTATCACG AGGCCCTTTC GTCTTCAAG	Sequencing Primer
BBP3086	GCTATCGTGA CCTTGATAAC GGCTGAC	Sequencing Primer
BBP3091	CTTGACGAGT TCTTCTGAGC GGG**ATC**CTGG GGTTCGCGGA ATTAATTC	Add BamHI to pEB001
BBP3092	GAATTAATTC CGCGAACCCC AGGATCCCGC TCAGAAGAAC TCGTCAAG	Add BamHI to pEB001
BBP3093	GGTTAATTAA GGGCTGCAGG **G**AT**C**CGATAT CAAGCTTATC G	Add BamHI to pBB295
BBP3094	CGATAAGCTT GATATCGGAT CCCTGCAGCC CTTAATTAAC C	Add BamHI to pBB295
BBP3152	CTTTTCGGGG TTAATTAAGG G**GGTACC**GAA TTCGATATCA AGCTTATCGA TAC	Add KpnI to pBB295
BBP3153	GTATCGATAA GCTTGATATC GAATTCGGTA CCCCCTTAAT TAACCCCGAA AAG	Add KpnI to pBB295

### 2.2 Growth of donor and recipient bacterial strains

*Azotobacter vinelandii* DJ (ATCC BAA‐1303) was grown on Burk's (B) medium (Dos Santos, 2011). *Gluconacetobacter diazotrophicus* PA1 5 was grown on GADN medium (Fisher & Newton, 2005; Schwister et al., 2022). *Klebsiella grimontii* M5aL, *Azoarcus olearius* DQS‐4, *Rhodobacter sphaeroides* 2.4.1, *Marinobacter aquaeolei* VT8 and *Escherichia coli* MG1655 were grown on lysogeny broth (LB) medium. Plates of LB were supplemented with 100 μL of 5 mg/mL 2,6‐diaminopimelic acid (DAP) when growing *E. coli* WM3064. Recipient strains were cultured in liquid medium before the introduction of the transposon.

### 2.3 Introduction of deoxyviolacein piggyback transposons to model bacteria

Recipient strains were conjugated with transposons following a protocol similar to that used for conjugation with *E. coli* WM3064 in our laboratory (Barney et al., 2015; Knutson et al., 2021). Recipient strains were first grown in liquid medium for a sufficient time to ensure that cells were in a planktonic form, especially for strains that formed biofilms, hard colonies or extensive exopolysaccharides. *E. coli* WM3064 containing the desired plasmid was scraped using a loop from a 1‐day‐old LB plate grown at 30°C containing the specific antibiotic and supplemented with DAP and transferred to a 1.5 mL microfuge tube to obtain ~100 μL volume of cells. The recipient strains were centrifuged for 1 min at ~10,000*g* to obtain a similar volume of cells. Both cell pellets were resuspended in 0.5 mL of growth medium, and depending on the growth rate of the recipient cells, we mixed different ratios of the two strains in ~100 μL volume. For example, we combined 5 μL of the *E. coli* WM3064 strain containing plasmid with 100 μL of *G. diazotrophicus* and mixed thoroughly before spotting them as 9 evenly spaced drops onto GADN medium supplemented with DAP. In each case, we confirmed that the *E. coli* WM3064 strain would grow on the specific medium before combining the two strains. During the conjugation and outgrowth procedures, no antibiotic was included on the plates. After the cultures had grown overnight, we scraped 3 spots per plate and grew them in 50 mL of the desired liquid medium. The liquid medium lacked DAP, resulting in a counter‐selection of the *E. coli* WM3064 and the enrichment of the recipient strain, and allowed the cells to recover before challenging with antibiotics. We then plated various aliquots of the liquid cultures onto solid medium with antibiotic (but devoid of DAP) for selection. Colonies were screened to determine the number of transposon‐integrated colonies and to identify specific colonies with sufficient pigment for the desired applications (Barney et al., 2015; Knutson et al., 2021). The methodology and ratios of donor and recipient strains had to be adjusted on a case‐by‐case basis for each strain to optimize the conjugation conditions.

### 2.4 Confirmation of genome integration sites

For specific mutations that yielded a desired amount of pigment, the site where the transposon had integrated and the direction of the insert was determined. This was accomplished by first purifying the individual colony by repeated passage on selective medium. Then genomic DNA was collected using the Quick‐DNA Fungal/Bacterial Miniprep Kit (Zymo Research). Genomic DNA was digested using the PstI restriction endonuclease, to cut the genomic DNA into suitable fragments. The DNA was then purified using the DNA Clean and Concentrator‐25 (Zymo Research) kit, and ligated to generate circular fragments suitable for PCR. Primers BBP3086 and either BBP1282 (for pBB327) or BBP1176 (for pBB328), were used to perform PCR and confirm a suitable DNA product for sequencing. Positive PCR products were purified using the DNA Clean and Concentrator‐25 kit, then sent for Sanger sequencing using primer BBP3086, which provided the insert location and direction in which the transposon had integrated into the genome.

### 2.5 Assessment of growth rate for specific isolates

To determine if there was a significant growth penalty for selected strains used in further tracer or biosensor studies, strains were streaked pure and several single colonies were used to inoculate each strain into a 50 mL culture of the medium described above for each strain. For each strain, control of the parent strain was also included so that the deoxyviolacein‐producing strain could be compared to its parent. Optical density was measured at 600 nm, once the strain had reached a starting density of approximately OD_600_ = 0.1. An additional measurement was made when cells had reached an OD_600_ above 1.0, but while the cells were still in exponential growth. In each case, the analysis was performed in triplicate and was used to determine the growth rate or doubling time. In most cases, colonies were selected with sufficient pigment levels and colony sizes that were comparable to surrounding non‐pigmented strains.

### 2.6 Deoxyviolacein quantification

Deoxyviolacein was quantified using a method similar to what has been previously described (Wang et al., 2009). Five milliliters of liquid culture was pelleted at >10,000*g* for 1 min and the supernatant was removed. The cell pellet was then rinsed using distilled water and pelleted again at >10,000*g* for 1 min. The cell pellet was resuspended in 5 mL of ethanol and sonicated for 5 min at 200 W using a Misonix XL‐2000 Series sonicator probe, then the cell debris was pelleted again at >10,000*g* for 1 min and the supernatant was measured for deoxyviolacein at 570 nm.

### 2.7 Infiltration of leaves and recovery of bacteria

Leaves of 4‐week‐old *Nicotiana benthamiana* plants were grown with adequate water and infiltrated with a pigment‐producing isolate of *Klebsiella grimontii* M5aL by a modification of a previously described method (Zhang et al., 2011). Briefly, bacteria were grown overnight in LB medium with 15 µg/mL of tetracycline. Cells from 2 mL of the culture were pelleted by centrifugation at 3500*g* for 5 min and the cells were resuspended in 20 mL of 0.25X MS medium. The resuspended bacteria were infiltrated into leaves through the stomates of the lower leaf surface using a needle‐less 1 mL syringe. To quantify the endophytes, the infiltrated leaves were surface sterilized in 10% bleach and 0.1% Tween 20 for 20 min and washed with distilled water. The infiltrated portion of leaves was ground in 500 µL of sterile ddH_2_O using a Bullet Blender to release endophytes. Serial dilutions of the solution remaining following grinding were plated on LB plates and endophytes were grown at 30°C.

### 2.8 Construction of an improved nitrogen‐compound biosensor strain of *A. vinelandii*

*A. vinelandii* is a model microbe for the study of aerobic nitrogen fixation. Testing mutants for extracellular nitrogen compound production is important to developing strains with improved biofertilizer potential. A prior effort developed a biosensor strain to indicate the presence of extracellular nitrogen compounds from mutant screens, but required the addition of 5‐bromo‐4‐chloro‐3‐indolyl β‐d‐galactopyranoside (X‐Gal) to plates (Barney et al., 2015). Developing a deoxyviolacein‐based biosensor would alleviate the need to add X‐Gal and potentially result in an improved reporter system. *A. vinelandii* strain AZBB120 lacks urease and the ammonium transporter AmtB (Eberhart et al., 2016). AZBB120 was transformed with pPCRNH3‐21 (Barney et al., 2015) to replace the genes coding the nitrogenase regulatory proteins NifL and NifA with a tetracycline cassette, yielding strain AZBB202, which can no longer fix nitrogen (Table 3). AZBB202 was then conjugated with pBB327 to select colonies with elevated deoxyviolacein production, yielding AZBB741. AZBB741 is unable to fix nitrogen diazotrophically or effectively utilize urea, but grows and produces deoxyviolacein pigment in the presence of nitrogen substrates such as nitrate and elevated ammonium, making it suitable to identify other microbes that produce specific extracellular nitrogen compounds.

**Table 3:** Bacterial strains used in this study.

Microbial strain	Genetic features	Insertion (if applicable)	Parent strain/Reference
*Azotobacter vinelandii* DJ	Wild‐type		
*Gluconacetobacter diazotrophicus* PA1 5	Wild‐type		
*Klebsiella grimontii* M5aL	Wild‐type		
*Azoarcus olearius* DQS‐4	Wild‐type		
*Escherichia coli* MG1655	Wild‐type		
GABB015	*vioABCE* transposon insert (pBB328)	Gdia_0657 (backwards)	*G. diazotrophicus* Pa1 5
AZBB202	Δ*ureABC*, Δ*amtB, nifLA::tet* ^*R*^		AZBB120 (Eberhart et al., 2016)
AZBB741	Δ*ureABC*, Δ*amtB, nifLA::tet* ^*R*^, *vioABCE* transposon insert (pBB327)	Avin_33840 (backwards)	AZBB202 (This study)
KGBB001	*vioABCE* transposon insert (pBB328)	BWI76_19555 (backwards)	*K. grimontii* M5aL
AOBB001	*vioABCE* transposon insert (pBB328)	Dqs_2299 (upstream)	*A. olearius* DQS‐4

## 3 RESULTS

### 3.1 Application of the deoxyviolacein‐based transposon system

**Figure 2:**
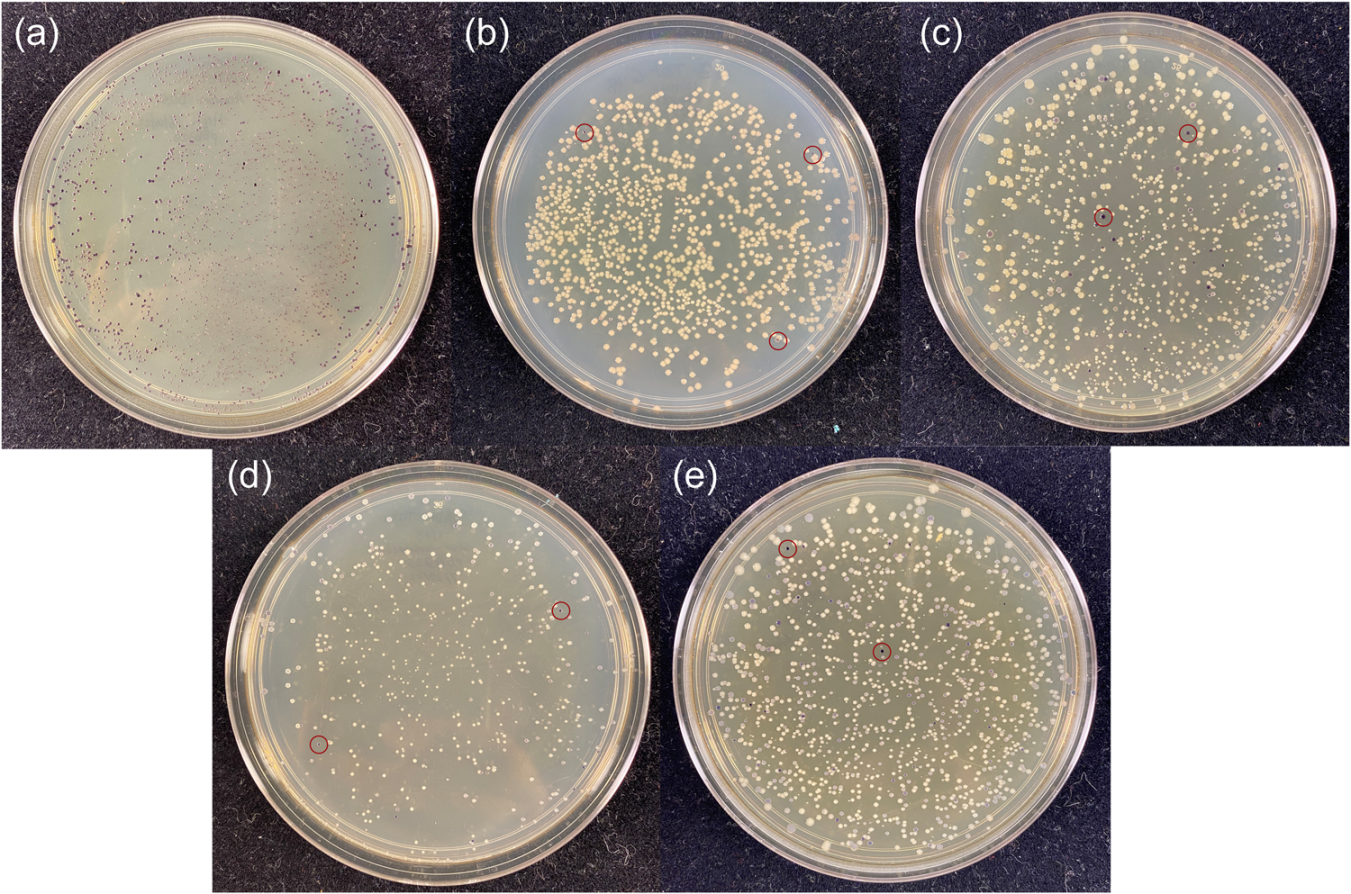
Transposon insertions for various bacteria. Shown above are examples of strains conjugated with the deoxyviolacein‐based transposon. Strains shown include *Azoarcus olearius* (a), *Azotobacter vinelandii* (b), *Escherichia coli* (c), *Gluconacetobacter diazotrophicus* (d) and *Klebsiella grimontii* (e). In plates with a low number of pigment‐producing colonies, several pigment‐producing colonies have been highlighted with a red circle on each plate (b–e).

The plasmids pBB327 and pBB328 were constructed with either a kanamycin selection marker or a tetracycline selection marker, respectively. As an initial test case, we conjugated *G. diazotrophicus* with either plasmid and looked for colonies with a suitable amount of pigment. Both plasmids yielded several colonies (approximately 10 per plate of 500 colonies, Figure 2) with a suitable amount of pigment (sufficient to confirm color development within 3 days of growth on GADN medium). Several colonies were selected and passaged on plates with antibiotics, and then passaged to plates without the antibiotic, to determine if the pigmented phenotype would be lost in the absence of antibiotic. In each case, there was minimal evidence of strain instability during each passage. Genomic locations for the inserts were determined to confirm that the resulting colonies had incorporated the transposon into the genome. Seven colonies were sequenced to determine the location of the TA insertion and were confirmed to contain a genomic insertion located at a specific TA site (insertions found in Gdia_0263, Gdia_0657, Gdia_1202, Gdia_1182, Gdia_2010, Gdia_2094 and Gdia_2169) (Giongo et al., 2010), indicating that the Mariner transposon functioned as designed with the *vioABCE* operon inserted into a random TA site within the genome. In addition to experiments with *G. diazotrophicus*, we also introduced the transposon into *Escherichia coli*, *Azotobacter vinelandii*, *Azoarcus olearius* and *Klebsiella grimontii*. The list of recipient strains is not intended to be an exhaustive selection of potential hosts. Based on the plasmid backbone, we believe it is reasonable to assume that any strain that can be conjugated with the Mariner transposon is also suitable for yielding transposon libraries with these plasmids, and there is a significant body of literature reporting successful transformation with the Mariner transposon for Tn‐seq experiments (Christen et al., 2011; Hutchison et al., 2019; Poulsen et al., 2019; Rosconi et al., 2016; van Opijnen et al., 2009; Zomer et al., 2012). While there is some evidence that violacein and deoxyviolacein may have antibacterial properties, this also appears to be strain‐specific, and we did not observe evidence of this in the five strains listed above.

**Figure 3:**
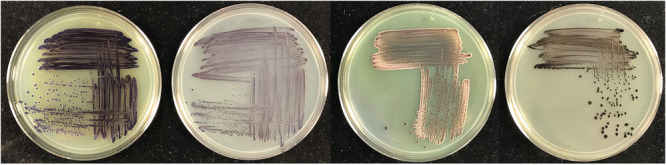
Shown are selected isolates of bacteria derived from plates similar to those shown in Figure 2 that express suitable amounts of deoxyviolacein for downstream studies. Strains shown are (from left to right), *Klebsiella grimontii*, *Gluconacetobacter diazotrophicus*, *Azotobacter vinelandii*, and *Azoarcus olearius*. These high pigment‐producing isolates enable easy identification from other bacteria. The strains shown represent a range of pigment production to illustrate the varying levels of deoxyviolacein that can be produced in different bacteria.

Shown in Figure 2 are several representative plates of recipient strains that were tested for transposon mutagenesis and pigment production. The plates were selected to illustrate relative differences in colony pigment levels and are not intended to be comprehensive. In many cases, several plates were prepared from each recipient to select an ideal mutant. For four of the recipient strains shown in Figure 2, only a small number of colonies per plate yielded significant amounts of pigment for further evaluation. However, for *A. olearius*, a much larger percentage of colonies showed substantial pigment production, indicating that the transposon may give varying degrees of pigment‐producing colonies depending on the recipient strain. For the remaining strains, these results confirm that the transposon is not carrying a strong internal promoter system, and is relying on suitable transcriptional initiators that are internal to the genome. Interestingly, when TA sites were located for the specific clones shown in Figure 3 (and listed above for *G. diazotrophicus*), the upstream element responsible for transcription of the *vioABCE* operon was not always immediately evident. Differential expression in different strain backgrounds is expected, and users should evaluate for themselves conditional versus constitutive expression in their target strain of interest.

To confirm the stability of individual strains, colonies were passaged on plates for several generations to obtain clean colonies and assess the stability of the pigment‐producing phenotype. This was done both in the presence of antibiotic and in many cases, once isolated, without the inclusion of the antibiotic. In some rare cases, a colony would illustrate a propensity to develop a small number of revertants in each passage, manifesting as a loss of pigment in several colonies on the plates. In these cases, the issue was resolved by either selecting another initial colony or by using the alternative plasmid and antibiotic selection to generate alternative transformants. In each of the cases shown in Figure 3, strains could be passaged on fresh plates without antibiotic pressure and exhibited little evidence of revertants or loss of the pigment‐producing phenotype. This again must be assessed on a case‐by‐case basis. Since we generally selected strains that grew at rates similar to the background strain, based on the sizes of the colonies and balanced production of the pigments, we believe that we also selected strains with a lower growth defect and comparable fitness level to the parent strains, as described below. This would indicate a lower selective pressure for the strains to revert or develop additional mutations that disrupted the pigment production.

### 3.2 Measurement of strain growth rate

As described in the methods, we were careful to select strains of pigmented tracers or biosensors that grew at rates similar to surrounding non‐pigmented colonies (as assessed by colony size). For this reason, we assumed that growth rates for these strains would be similar to the starting parental strains. For strain KGBB001 (Table 3), the growth rate was slightly higher (~15%) than for the wild‐type *K. grimontii* strain that served as the parent strain. For strain AZBB741, the growth rate was also slightly higher (~20%) than the parent strain of AZBB202. Both AZBB202 and AZBB741 were grown in B medium supplemented with 20 mM sodium nitrate, as these strains have a disruption in the *nifLA* operon, resulting in the disruption of the diazotrophic phenotype. Finally, GABB015 had a slightly slower growth rate (~30%) than the wild‐type *G. diazotrophicus* strain that served as the parent strain. In the case of *K. grimontii* and *G. diazotrophicus*, these strains were grown on a rich medium that should contain a source of tryptophan, while for *A. vinelandii* strains AZBB202 and AZBB741, the strains were supplemented with an external nitrogen source, such that none of these three strains were grown under diazotrophic conditions. Additionally, while the growth rates were relatively similar for all three pigment‐producing strains, the lag phase for each of these strains was slightly longer.

### 3.3 Studies of leaf endophytes

**Figure 4:**
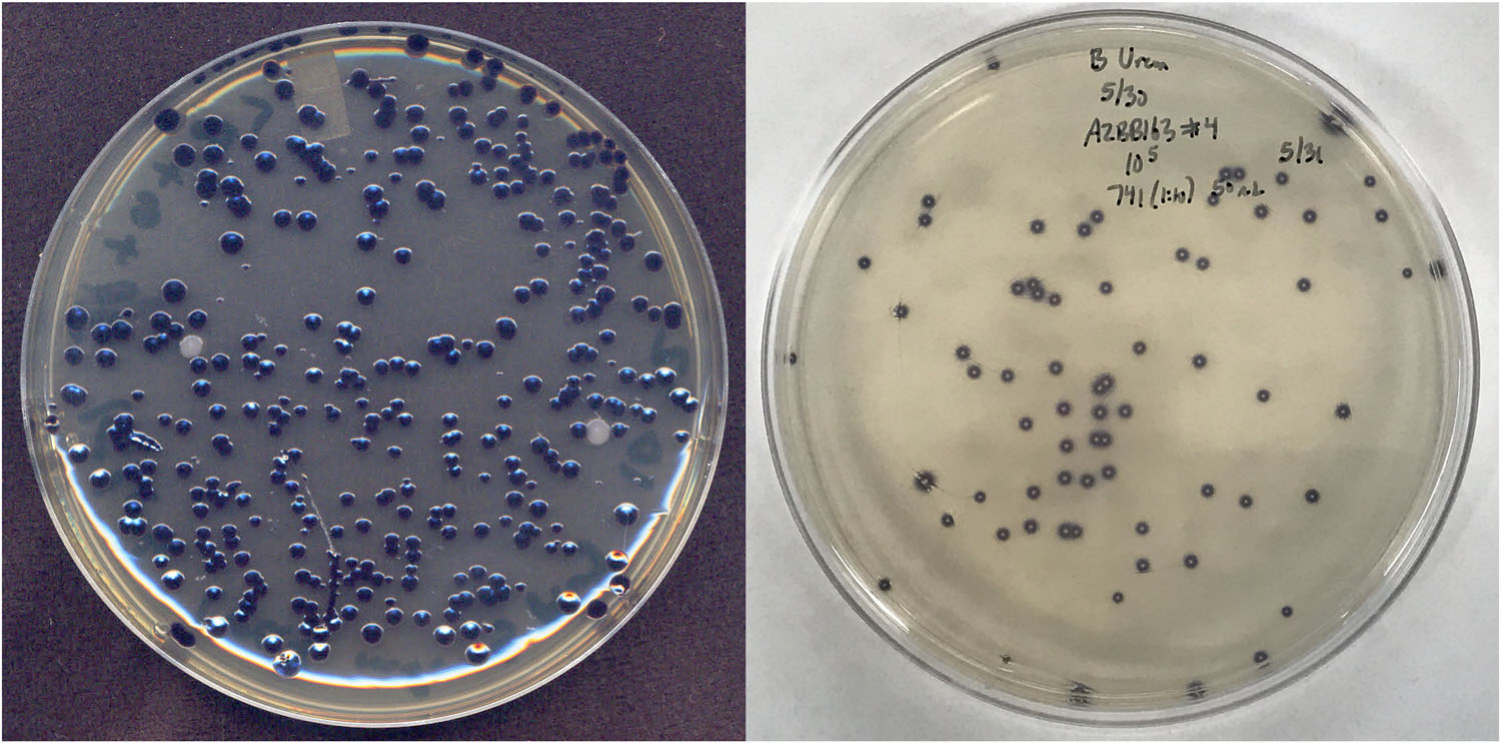
Example applications of deoxyviolacein‐modified strains. Shown on the left is a sample obtained following serial dilution of *Klebsiella grimontii* endophytes recovered from infiltrated leaves of *Nicotiana benthamiana* seven days after infiltration. The *K. grimontii* strain used to infiltrate these leaves expresses deoxyviolacein as a result of the pBB328 vector, allowing differentiation between the endophytes infiltrated into the strain and natural endophytes or contaminants. Light reflection on colonies near the bottom of the image is an artefact related to image collection. All pigmented colonies showed an even distribution of pigment over the entire colony. Shown on the right is a plate covered with a lawn of the nitrogen‐excreting biosensor strain *A. vinelandii* AZBB741 constructed here and plated together with a sample from a serial dilution of the extracellular nitrogen compound producing strain AZBB163 (Barney et al., 2015). Colonies yielding increased extracellular ammonium are identified by the purple halo resulting from the non‐diazotrophic AZBB741. Negative controls and images under a variety of conditions and at different magnifications are provided in the Appendix. This approach enables the identification of colonies that produce extracellular nitrogen compounds (surrounded by a purple halo).

Several colonies of the endophytes *K. grimontii* and *G. diazotrophicus* with the desired pigment‐producing phenotype were selected for studies with various plant hosts and were confirmed to maintain the pigmented phenotype even after several weeks in association with a plant host as an endophyte. Importantly, while in the presence of the plants, no antibiotic pressure was applied to the bacteria. At the end of these experiments, bacterial cells were plated back onto the specific selective medium with the specific antibiotic and screened for pigment production, indicating that these new strains could function effectively as suitable sensors in recovery experiments to distinguish them from other bacteria and determine numbers of viable cells recovered from leaves (Figure 4), roots or other plant organs. Figure 4 shows an example of one such experiment. The pigmented KGBB001 strain could be easily differentiated from other natural endophytes (purple vs. white colonies), which could also be differentiated based on differences in colony morphology.

### 3.4 Constructing an improved nitrogen‐compound biosensor strain

A strain of *A. vinelandii* containing deletions in the ammonium transporter and urease genes and a disruption in *nifLA* nitrogen regulator genes (AZBB202) was further conjugated with the deoxyviolacein transposon to isolate a strain with elevated pigment production (AZBB741). This strain is no longer diazotrophic, relying upon exogenous nitrogen sources for growth. This strain was used to plate serial dilutions of the elevated extracellular nitrogen compound‐producing strain AZBB163 (Barney et al., 2015) onto a lawn of AZBB741 to rapidly scan individual colonies of AZBB163 from elevated extracellular nitrogen compound production (Figure 4). The strains that are still producing elevated levels of extracellular nitrogen compounds are easily identified by the halo of purple‐pigmented AZBB741 growing immediately adjacent to them. This can be used to evaluate strains that are producing elevated ammonium and determine the potential loss of the phenotype in certain populations, if it had occurred.

## 4 DISCUSSION

The primary motivator for developing a deoxyviolacein‐based transposon system in our laboratory was to generate stable mutant strains that could be easily differentiated by the presence of the purple deoxyviolacein pigment (Kittleson et al., 2012). A prerequisite to applying this transposon system is that the deoxyviolacein pigment is not excessively toxic in the recipient strain. Both violacein and deoxyviolacein are proposed to have antibacterial and antifungal properties, though this is not universal for all microbes, and there are differences for each pigment. For example, deoxyviolacein was found to be less toxic as an antibacterial to *Staphylococcus aureus* and *Bacillus megaterium* than violacein (Wang et al., 2012), while deoxyviolacein was found to be a more potent antifungal than violacein in a number of target strains. Many other bacteria showed minimal susceptibility to deoxyviolacein (Wang et al., 2012). In this study, we demonstrated the ability to introduce pigment production in five Gram‐negative strains, though we were unable to obtain any colonies for *Marinobacter aquaeolei*. One potential limitation may also be related to the ability of the strains to be conjugated by the Mariner transposon.

There are many approaches one could take to generate stable strains to yield this pigment, including targeting genes to a specific location and swapping the deoxyviolacein operon in place of a non‐essential gene along with an antibiotic cassette for selection. However, this may require several rounds of design, build and testing to target the operon to a suitable promoter system that yields the desired amount of pigment, with a requirement to first construct a suitable vector to direct the genes to the target location. By inserting a transposon‐based cassette randomly throughout the genome, one can simply select a modified strain with the desired amount of pigment (Figure 2), and streak several times to determine if the strain is stable and has the desired strain characteristics when isolated from a plate of several hundred colonies (Figure 3). Levels of visible pigment (Figure 3) on plates for many of these mutants are similar to what is found in the native strain of *C. violaceum* (Devescovi et al., 2017). Transposon inserts that are detrimental to the cell, by either disrupting essential genes or producing levels of deoxyviolacein‐based pigments that might be toxic to the cell (Durán et al., 2007), will not survive among the larger library and are easily avoided. Inserts that lead to slower growth can be qualitatively assessed by comparing the size of colonies versus other mutant colonies on the plate, and then determining if the slower growth rate potentially outweighs the limitations due to improved pigment production. Indeed, many of the colonies generating increasing amounts of pigment in our initial screening of plates were accompanied by some degree of growth defect manifesting itself as smaller colony size compared to neighboring colonies. Inserts with insufficient pigment can also quickly be assessed and entirely avoided. In this manner, one can rapidly screen several thousand colonies simultaneously with little additional effort (Figure 2), and select a number of ideal colonies for further characterization (Figure 3). In theory, any strain that can be transformed with the Mariner transposon should be able to be screened using these simple vectors, and with minimum effort, generally yielding a sufficient clone in under 2 weeks (depending on the growth rate of the recipient strain).

The *lacZ* or β‐glucuronidase genes are commonly used to generate pigments within specific colonies related to a desired phenotype or the lack of the phenotype (Hirt, 1991; Ullmann et al., 1967; Vieira & Messing, 1982). These often require the addition of a precursor compound such as X‐Gal to solid growth medium (Horwitz et al., 1964), which can be costly, can degrade over time, or can generate some undesired background color, especially if strains carry a native *lacZ* homolog. One feature of the *vioABCE* gene operon is that the pigment is produced from intracellular tryptophan (Durán et al., 2007), which should be reasonably available in most strains. For some species, the pigment yield could be increased by supplementing tryptophan into the growth medium, but this would be dependent on a suitable means to transport tryptophan into the cell, and we generally avoided additional supplementation of tryptophan in selecting suitable candidate colonies. If the desired recipient strain is already a violacein or deoxyviolacein producer or produces other purple pigments, then this would limit the application of this system in those strains. Since there are very few bacteria that generate deoxyviolacein or other purple pigments, this system could be applied to a large number of bacterial species to better track the fate of the bacteria in different experiments, as was done here in endophyte studies, or to determine the numbers or fractions of cells of the specific strain in a complex mixture of microbes, such as a natural community or in binary cultures. It also enables the development of strains that can serve as a biosensor of a specific metabolite (such as nitrogen compounds) in combination with other strains that produce that metabolite, as shown in Figure 4 for *A. vinelandii*, a diazotroph that can be altered to produce extracellular ammonium (Barney et al., 2015; Plunkett et al., 2020). By constructing a strain of *A. vinelandii* incapable of fixing nitrogen and incorporating the deoxyviolacein transposon system into that strain, we identified a strain that can be used to screen for elevated nitrogen compound production of *A. vinelandii* mutants without the need to provide X‐Gal, as was previously employed (Barney et al., 2015).

We can imagine additional applications and experiments as well. For instance, if one wanted to find a gene or segment of the genome that reports the presence of a specific compound (e.g., galactose), then one could first isolate a library on a medium containing galactose, and streak colonies that produced pigment in the presence of galactose. Isolates could then be passaged to a medium devoid of galactose to search for an isolate that loses color when galactose is not present. The final strain could be used as a reporter for galactose, or to identify genes upregulated in the presence of galactose.

As part of our effort, we generated deoxyviolacein‐positive transposon mutants within a number of bacteria that are known to function as endophytes within plants. These included *Gluconacetobacter diazotrophicus* (Bertalan et al., 2009; Gillis et al., 1989; Giongo et al., 2010), *Azoarcus olearius* (Chen et al., 2013; Faoro et al., 2017) and *Klebsiella grimontii* (Pengra & Wilson, 1958; Stewart et al., 1967; Yu et al., 2018). Some strains yielded very few mutants, including *Azospirillum brasilense* (Pankievicz et al., 2015) and *Azoarcus communis* (Reinholdhurek et al., 1993). We also tested *Azotobacter vinelandii* and *Escherichia coli*. We were unable to obtain any mutants with *Marinobacter aquaeolei* (Huu et al., 1999). The two different plasmids constructed allow for selection with either tetracycline or kanamycin.

In many cases, strains that yielded high pigment levels were tracked to TA sites with no obvious upstream genetic elements that might be responsible for elevated expression of the *vioABCE* operon. We view this as a benefit, since this may help to identify nonobvious sites within the genome that one might not select to develop expression systems based on an inherent conceptualization of ideal expression sites. Once several sites are determined using this protocol, an individual could design target vectors to insert other genes containing a suitable RNA binding site (or use the GGAGGTAAAAAAA sequence that was engineered into the *vioABCE* synthetic operon) to then engineer suitable vectors for transferring other genes into the recipient strain in the future.

Violacein production in the natural host strain of *Chromobacterium violaceum* is accomplished by the *vioABCDE* operon (August et al., 2000), but our construct is based on the synthetic operon provided by Christopher Anderson that only contains the *vioABCE* genes (Kittleson et al., 2012). In this synthetic construct, each of the genes contains the same RBS site upstream of the genes. This strain produces the deoxyviolacein pigment, which has an absorbance feature at 570 nm that can be used to easily quantify approximate amounts of the deoxyviolacein pigment.

Many alternative methods to label bacteria with easily identifiable markers exist. Perhaps the most common is the application of fluorescent proteins such as green fluorescent protein (Chalfie et al., 1994). These methods are dependent and limited to some degree by the quantities of protein that can be produced and the rates of protein degradation within the cell. Additionally, many common fluorescent proteins require molecular oxygen, making them difficult for applications with anaerobic bacteria (Ko et al., 2020; Landete et al., 2015). Labels that produce a pigment through an enzymatic reaction of a common metabolite have the potential to yield far greater molar quantities of the pigment. Alternative pigments produced from amino acids include indigoidine produced from glutamine (Xie et al., 2017) and betalain produced from tyrosine (He et al., 2020).

In summary, we have demonstrated the successful application of a deoxyviolacein‐based piggyback transposon vector for generating pigment‐producing bacteria in a variety of important bacterial hosts, and serve as a method to track strains introduced into complex communities or as a biosensor for extracellular nitrogen compounds. The pigment production could have further application for random introduction into inducible promoters that are either known or unknown, and to serve as a sensor for an extensive number of metabolites. This system could have a much broader potential use to other laboratories and be an important tool for rapidly generating pigment‐producing clones in other bacterial hosts, assuming these do not have a native violacein system or produce a similar colored pigment and are not affected by the potential antibacterial properties of the pigment with certain strains. We have provided the plasmids containing these transposons through Addgene, so they can be easily accessed by other interested laboratories, and continue to use these constructs in tracer studies in our laboratory.

## AUTHOR CONTRIBUTIONS

**Benjamin R. Dietz**: Investigation (equal); methodology (equal); validation (equal); writing—original draft (equal). **Tyler J. Nelson**: Investigation (equal); Methodology (equal); Writing—review & editing (equal). **Neil E. Olszewski**: Funding acquisition (equal); Investigation (equal); Methodology (equal); Writing—review & editing (equal). **Brett M. Barney**: Conceptualization (equal); Funding acquisition (equal); Investigation (equal); Methodology (equal); Supervision (equal); Visualization (equal); Writing—original draft (equal); Writing—review & editing (equal).

## CONFLICT OF INTEREST STATEMENT

None declared.

## ETHICS STATEMENT

None required.

## Figures and Tables

**Figure A1:**
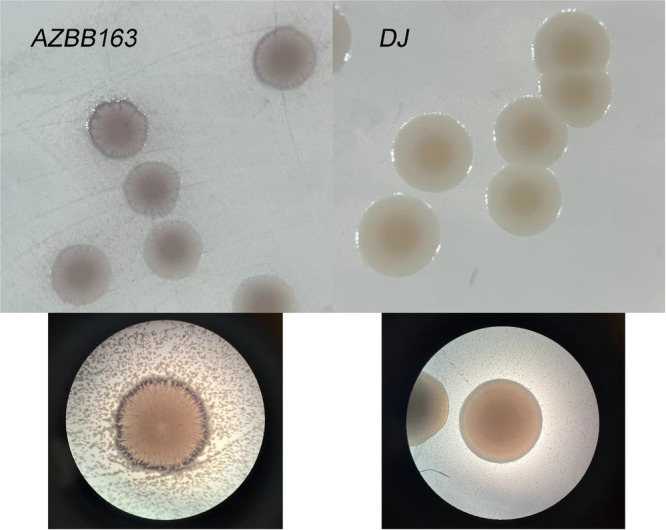
Shown on the top left is a section of a plate covered with a lawn of the nitrogen‐excreting biosensor strain *A. vinelandii* AZBB741 plated together with a sample from a serial dilution of the extracellular nitrogen compound producing strain AZBB163. The bottom left shows a higher degree of magnification under slightly different lighting for the single colony. Shown on the top right is a similar experiment with a lawn of *A. vinelandii* AZBB741 plated together with a sample from a serial dilution of the wild‐type DJ strain (negative control). These plates contained no extraneous nitrogen compounds and required a longer duration of time for the phenotype to become obvious.

**Figure A2:**
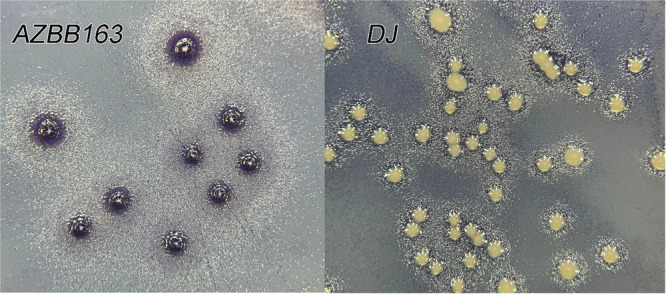
Shown on the left is a section of a plate covered with a lawn of the nitrogen‐excreting biosensor strain *A. vinelandii* AZBB741 plated together with a sample from a serial dilution of the extracellular nitrogen compound producing strain AZBB163. Shown on the right is a similar experiment with a lawn of *A. vinelandii* AZBB741 plated together with a sample from a serial dilution of the wild‐type DJ strain (negative control). These plates contained a small amount of background urea to initiate a slightly higher level of basal growth for the AZBB741 strain.

## Data Availability

Plasmids described here are publicly available through Addgene with ID 221532 and 221533.

## 1

(Figures A1 and A2)

Included are two appendix figures that provide examples of the positive and negative controls for the experiment shown in Figure 4 (right) to differentiate cells producing high levels of extracellular ammonium versus a wild‐type control of the *A. vinelandii* DJ strain, which produces very minimal extracellular nitrogen compounds.

## References

[mbo31425-bib-0001] August, P. R. , Grossman, T. H. , Minor, C. , Draper, M. P. , MacNeil, I. A. , Pemberton, J. M. , Call, K. M. , Holt, D. , & Osburne, M. S. (2000). Sequence analysis and functional characterization of the violacein biosynthetic pathway from *Chromobacterium violaceum* . Journal of Molecular Microbiology and Biotechnology, 2(4), 513–519. 11075927

[mbo31425-bib-0002] Barney, B. M. , Eberhart, L. J. , Ohlert, J. M. , Knutson, C. M. , & Plunkett, M. H. (2015). Gene deletions resulting in increased nitrogen release by *Azotobacter vinelandii*: Application of a novel nitrogen biosensor. Applied and Environmental Microbiology, 81(13), 4316–4328. 10.1128/AEM.00554-15 25888177 PMC4475869

[mbo31425-bib-0003] Bertalan, M. , Albano, R. , de Pádua, V. , Rouws, L. , Rojas, C. , Hemerly, A. , Teixeira, K. , Schwab, S. , Araujo, J. , Oliveira, A. , França, L. , Magalhães, V. , Alquéres, S. , Cardoso, A. , Almeida, W. , Loureiro, M. M. , Nogueira, E. , Cidade, D. , Oliveira, D. , … Ferreira, P. C. (2009). Complete genome sequence of the sugarcane nitrogen‐fixing endophyte *Gluconacetobacter diazotrophicus* Pal5. BMC Genomics, 10, 450. 10.1186/1471-2164-10-450 19775431 PMC2765452

[mbo31425-bib-0004] Brutinel, E. D. , & Gralnick, J. A. (2012). Anomalies of the anaerobic tricarboxylic acid cycle in *Shewanella oneidensis* revealed by Tn‐seq. Molecular Microbiology, 86(2), 273–283. 10.1111/j.1365-2958.2012.08196.x 22925268

[mbo31425-bib-0005] Chalfie, M. , Tu, Y. , Euskirchen, G. , Ward, W. W. , & Prasher, D. C. (1994). Green fluorescent protein as a marker for gene expression. Science, 263(5148), 802–805. 10.1126/science.8303295 8303295

[mbo31425-bib-0006] Chen, M. H. , Sheu, S. Y. , James, E. K. , Young, C. C. , & Chen, W. M. (2013). *Azoarcus olearius* sp nov., a nitrogen‐fixing bacterium isolated from oil‐contaminated soil. International Journal of Systematic and Evolutionary Microbiology, 63, 3755–3761. 10.1099/ijs.0.050609-0 23645022

[mbo31425-bib-0007] Christen, B. , Abeliuk, E. , Collier, J. M. , Kalogeraki, V. S. , Passarelli, B. , Coller, J. A. , Fero, M. J. , McAdams, H. H. , & Shapiro, L. (2011). The essential genome of a bacterium. Molecular Systems Biology, 7, 528. 10.1038/msb.2011.58 21878915 PMC3202797

[mbo31425-bib-0008] Devescovi, G. , Kojic, M. , Covaceuszach, S. , Cámara, M. , Williams, P. , Bertani, I. , Subramoni, S. , & Venturi, V. (2017). Negative regulation of violacein biosynthesis in *Chromobacterium violaceum* . Frontiers in Microbiology, 8, 349. 10.3389/fmicb.2017.00349 28326068 PMC5339254

[mbo31425-bib-0009] Durán, N. , Justo, G. Z. , Ferreira, C. V. , Melo, P. S. , Cordi, L. , & Martins, D. (2007). Violacein: Properties and biological activities. Biotechnology and Applied Biochemistry, 48, 127–133. 10.1042/ba20070115 17927569

[mbo31425-bib-0010] Eberhart, L. J. , Knutson, C. M. , & Barney, B. M. (2016). A methodology for markerless genetic modifications in *Azotobacter vinelandii* . Journal of Applied Microbiology, 120(6), 1595–1604. 10.1111/jam.13091 26854474

[mbo31425-bib-0011] Faoro, H. , Rene Menegazzo, R. , Battistoni, F. , Gyaneshwar, P. , do Amaral, F. P. , Taulé, C. , Rausch, S. , Gonçalves Galvão, P. , de los Santos, C. , Mitra, S. , Heijo, G. , Sheu, S. Y. , Chen, W. M. , Mareque, C. , Zibetti Tadra‐Sfeir, M. , Ivo Baldani, J. , Maluk, M. , Paula Guimarães, A. , Stacey, G. , … James, E. K. (2017). The oil‐contaminated soil diazotroph *Azoarcus olearius* DQS‐4^T^ is genetically and phenotypically similar to the model grass endophyte *Azoarcus* sp BH72. Environmental Microbiology Reports, 9(3), 223–238. 10.1111/1758-2229.12502 27893193

[mbo31425-bib-0012] Fisher, K. , & Newton, W. E. (2005). Nitrogenase proteins from *Gluconacetobacter diazotrophicus*, a sugarcane‐colonizing bacterium. Biochimica et Biophysica Acta (BBA) ‐ Proteins and Proteomics, 1750(2), 154–165. 10.1016/j.bbapap.2005.04.010 15925553

[mbo31425-bib-0013] Gillis, M. , Kersters, K. , Hoste, B. , Janssens, D. , Kroppenstedt, R. M. , Stephan, M. P. , Teixeira, K. R. S. , Dobereiner, J. , & De Ley, J. (1989). *Acetobacter diazotrophicus* sp. nov., a Nitrogen‐Fixing Acetic Acid Bacterium Associated with Sugarcane. International Journal of Systematic Bacteriology, 39(3), 361–364.

[mbo31425-bib-0014] Giongo, A. , Tyler, H. L. , Zipperer, U. N. , & Triplett, E. W. (2010). Two genome sequences of the same bacterial strain, *Gluconacetobacter diazotrophicus* PAl 5, suggest a new standard in genome sequence submission. Standards in Genomic Sciences, 2(3), 309–317. 10.4056/sigs.972221 21304715 PMC3035290

[mbo31425-bib-0015] Hayes, F. (2003). Transposon‐based strategies for microbial functional genomics and proteomics. Annual Review of Genetics, 37, 3–29. 10.1146/annurev.genet.37.110801.142807 14616054

[mbo31425-bib-0016] He, Y. , Zhang, T. , Sun, H. , Zhan, H. , & Zhao, Y. (2020). A reporter for noninvasively monitoring gene expression and plant transformation. Horticulture Research, 7(1), 152. 10.1038/s41438-020-00390-1 33024566 PMC7502077

[mbo31425-bib-0017] Hirt, H. (1991). A novel method for in situ screening of yeast colonies with the β‐glucuronidase reporter gene. Current Genetics, 20(5), 437–439. 10.1007/bf00317075 1807836

[mbo31425-bib-0018] Horwitz, J. P. , Chua, J. , Curby, R. J. , Tomson, A. J. , Da Rooge, M. A. , Fisher, B. E. , Mauricio, J. , & Klundt, I. (1964). Substrates for cytochemical demonstration of enzyme activity. I. some substituted 3‐Indolyl‐β‐d‐glycopyranosides. Journal of Medicinal Chemistry, 7(4), 574–575. 10.1021/jm00334a044 14221156

[mbo31425-bib-0019] Hutchison, C. A. , Merryman, C. , Sun, L. , Assad‐Garcia, N. , Richter, R. A. , Smith, H. O. , & Glass, J. I. (2019). Polar effects of transposon insertion into a minimal bacterial genome. Journal of Bacteriology, 201(19), e00185-19 10.1128/jb.00185-19 31262838 PMC6755753

[mbo31425-bib-0020] Huu, N. B. , Denner, E. B. , Ha, D. T. , Wanner, G. , & Stan‐Lotter, H. (1999). *Marinobacter aquaeolei* sp. nov., a halophilic bacterium isolated from a Vietnamese oil‐producing well. International Journal of Systematic Bacteriology, 49 Pt 2, 367–375. 10319457 10.1099/00207713-49-2-367

[mbo31425-bib-0021] Kittleson, J. T. , DeLoache, W. , Cheng, H. Y. , & Anderson, J. C. (2012). Scalable plasmid transfer using engineered P1‐based phagemids. ACS Synthetic Biology, 1(12), 583–589. 10.1021/sb300054p 23656280 PMC3804010

[mbo31425-bib-0022] Knutson, C. M. , Pieper, M. N. , & Barney, B. M. (2021). Gene fitness of *Azotobacter vinelandii* under diazotrophic growth. Journal of Bacteriology, 203(24), e0040421. 10.1128/jb.00404-21 34570624 PMC8604073

[mbo31425-bib-0023] Ko, S. , Jeon, H. , Yoon, S. , Kyung, M. , Yun, H. , Na, J. H. , & Jung, S. T. (2020). Discovery of novel *Pseudomonas putida* flavin‐binding fluorescent protein variants with significantly improved quantum yield. Journal of Agricultural and Food Chemistry, 68(21), 5873–5879. 10.1021/acs.jafc.0c00121 32367716

[mbo31425-bib-0024] Landete, J. M. , Langa, S. , Revilla, C. , Margolles, A. , Medina, M. , & Arqués, J. L. (2015). Use of anaerobic green fluorescent protein versus green fluorescent protein as reporter in lactic acid bacteria. Applied Microbiology and Biotechnology, 99(16), 6865–6877. 10.1007/s00253-015-6770-3 26129953

[mbo31425-bib-0025] van Opijnen, T. , Bodi, K. L. , & Camilli, A. (2009). Tn‐seq: High‐throughput parallel sequencing for fitness and genetic interaction studies in microorganisms. Nature Methods, 6(10), 767–772. 10.1038/nmeth.1377 19767758 PMC2957483

[mbo31425-bib-0026] Pankievicz, V. C. S. , do Amaral, F. P. , Santos, K. F. D. N. , Agtuca, B. , Xu, Y. , Schueller, M. J. , Arisi, A. C. M. , Steffens, M. B. R. , de Souza, E. M. , Pedrosa, F. O. , Stacey, G. , & Ferrieri, R. A. (2015). Robust biological nitrogen fixation in a model grass‐bacterial association. The Plant Journal, 81(6), 907–919. 10.1111/tpj.12777 25645593

[mbo31425-bib-0027] Pengra, R. M. , & Wilson, P. W. (1958). Physiology of nitrogen fixation by *Aerobacter aerogenes* . Journal of Bacteriology, 75(1), 21–25. 10.1128/jb.75.1.21-25.1958 13513555 PMC290026

[mbo31425-bib-0028] Plunkett, M. H. , Knutson, C. M. , & Barney, B. M. (2020). Key factors affecting ammonium production by an *Azotobacter vinelandii* strain deregulated for biological nitrogen fixation. Microbial Cell Factories, 19(1), 107. 10.1186/s12934-020-01362-9 32429912 PMC7238568

[mbo31425-bib-0029] Poulsen, B. E. , Yang, R. , Clatworthy, A. E. , White, T. , Osmulski, S. J. , Li, L. , Penaranda, C. , Lander, E. S. , Shoresh, N. , & Hung, D. T. (2019). Defining the core essential genome of *Pseudomonas aeruginosa* . Proceedings of the National Academy of Sciences, 116(20), 10072–10080. 10.1073/pnas.1900570116 PMC6525520 31036669

[mbo31425-bib-0030] Reinholdhurek, B. , Hurek, T. , Gillis, M. , Hoste, B. , Vancanneyt, M. , Kersters, K. , & Deley, J. (1993). *Azoarcus* gen. nov., nitrogen‐fixing proteobacteria associated with roots of kallar grass (*Leptochloa fusca* (L.) Kunth), and description of two species, *Azoarcus indigens* sp. nov. and *Azoarcus communis* sp. nov. International Journal of Systematic Bacteriology, 43(3), 574–584. 10.1099/00207713-43-3-574

[mbo31425-bib-0031] Rosconi, F. , de Vries, S. P. W. , Baig, A. , Fabiano, E. , & Grant, A. J. (2016). Essential genes for in vitro growth of the endophyte *Herbaspirillum seropedicae* SmR1 as revealed by transposon insertion site sequencing. Applied and Environmental Microbiology, 82(22), 6664–6671. 10.1128/aem.02281-16 27590816 PMC5086560

[mbo31425-bib-0032] Rubin, E. J. , Akerley, B. J. , Novik, V. N. , Lampe, D. J. , Husson, R. N. , & Mekalanos, J. J. (1999). In vivo transposition of mariner‐based elements in enteric bacteria and mycobacteria. Proceedings of the National Academy of Sciences, 96(4), 1645–1650. 10.1073/pnas.96.4.1645 PMC15546 9990078

[mbo31425-bib-0033] Dos Santos, P. C. (2011). Molecular biology and genetic engineering in nitrogen fixation. Methods in Molecular Biology, 766, 81–92. 10.1007/978-1-61779-194-9_6 21833862

[mbo31425-bib-0034] Schwister, E. M. , Dietz, B. R. , Knutson, C. M. , Olszewski, N. E. , & Barney, B. M. (2022). *Gluconacetobacter diazotrophicus* gene fitness during diazotrophic growth. Applied and Environmental Microbiology, 88(23), 1–15. 10.1128/aem.01241-22 PMC9746312 36374093

[mbo31425-bib-0035] Stewart, W. D. P. , Fitzgerald, G. P. , & Burris, R. H. (1967). In situ studies on nitrogen fixation with the acetylene reduction technique. Science, 158(3800), 536. 10.1126/science.158.3800.536 17749121

[mbo31425-bib-0036] Ullmann, A. , Jacob, F. , & Monod, J. (1967). Characterization by in vitro complementation of a peptide corresponding to an operator‐proximal segment of the β‐galactosidase structural gene of *Escherichia coli* . Journal of Molecular Biology, 24(2), 339–343. 10.1016/0022-2836(67)90341-5 5339877

[mbo31425-bib-0037] Vieira, J. , & Messing, J. (1982). The pUC plasmids, an M13mp7‐derived system for insertion mutagenesis and sequencing with synthetic universal primers. Gene, 19(3), 259–268. 10.1016/0378-1119(82)90015-4 6295879

[mbo31425-bib-0038] Wang, H. , Jiang, P. , Lu, Y. , Ruan, Z. , Jiang, R. , Xing, X. H. , Lou, K. , & Wei, D. (2009). Optimization of culture conditions for violacein production by a new strain of *Duganella* sp B2. Biochemical Engineering Journal, 44(2–3), 119–124. 10.1016/j.bej.2008.11.008

[mbo31425-bib-0039] Wang, H. , Wang, F. , Zhu, X. , Yan, Y. , Yu, X. , Jiang, P. , & Xing, X. H. (2012). Biosynthesis and characterization of violacein, deoxyviolacein and oxyviolacein in heterologous host, and their antimicrobial activities. Biochemical Engineering Journal, 67, 148–155. 10.1016/j.bej.2012.06.005

[mbo31425-bib-0040] Xie, Z. , Zhang, Z. , Cao, Z. , Chen, M. , Li, P. , Liu, W. , Qin, H. , Zhao, X. , Tao, Y. , & Chen, Y. (2017). An external substrate‐free blue/white screening system in *Escherichia coli* . Applied Microbiology and Biotechnology, 101(9), 3811–3820. 10.1007/s00253-017-8252-2 28352998

[mbo31425-bib-0041] Yu, Z. , Li, S. , Li, Y. , Jiang, Z. , Zhou, J. , & An, Q. (2018). Complete genome sequence of N2‐fixing model strain *Klebsiella* sp. nov. M5al, which produces plant cell wall‐degrading enzymes and siderophores. Biotechnology Reports, 17(6–9), 6–9. 10.1016/j.btre.2017.11.006 29234606 PMC5723360

[mbo31425-bib-0042] Zhang, Z. L. , Ogawa, M. , Fleet, C. M. , Zentella, R. , Hu, J. , Heo, J. O. , Lim, J. , Kamiya, Y. , Yamaguchi, S. , & Sun, T. (2011). Scarecrow‐like 3 promotes gibberellin signaling by antagonizing master growth repressor DELLA in arabidopsis. Proceedings of the National Academy of Sciences, 108(5), 2160–2165. 10.1073/pnas.1012232108 PMC3033277 21245327

[mbo31425-bib-0043] Zomer, A. , Burghout, P. , Bootsma, H. J. , Hermans, P. W. M. , & van Hijum, S. A. F. T. (2012). Essentials: Software for rapid analysis of high throughput transposon insertion sequencing data. PLoS One, 7(8), e43012. 10.1371/journal.pone.0043012 22900082 PMC3416827

